# Effect of miscarriage history on maternal-infant bonding during the first year postpartum in the First Baby Study: a longitudinal cohort study

**DOI:** 10.1186/1472-6874-14-83

**Published:** 2014-07-15

**Authors:** Cara Bicking Kinsey, Kesha Baptiste-Roberts, Junjia Zhu, Kristen H Kjerulff

**Affiliations:** 1 College of Nursing, The Pennsylvania State University, 90 Hope Drive, Hershey, PA 17033, USA; 2 College of Medicine, Department of Public Health Sciences, The Pennsylvania State University, Hershey, PA, USA; 3 College of Medicine, Departments of Public Health Sciences and Obstetrics and Gynecology, The Pennsylvania State University, Hershey, PA, USA

**Keywords:** Maternal-infant bonding, Perinatal loss, Miscarriage, Parenting, Postpartum Bonding Questionnaire

## Abstract

**Background:**

Miscarriage, the unexpected loss of pregnancy before 20 weeks gestation, may have a negative effect on a mother’s perception of herself as a capable woman and on her emotional health when she is pregnant again subsequent to the miscarriage. As such, a mother with a history of miscarriage may be at greater risk for difficulties navigating the process of becoming a mother and achieving positive maternal-infant bonding with an infant born subsequent to the loss. The aim of this study was to examine the effect of miscarriage history on maternal-infant bonding after the birth of a healthy infant to test the hypothesis that women with a history of miscarriage have decreased maternal-infant bonding compared to women without a history of miscarriage.

**Methods:**

We completed secondary analysis of the First Baby Study, a longitudinal cohort study, to examine the effect of a history of miscarriage on maternal-infant bonding at 1 month, 6 months, and 12 months after women experienced the birth of their first live-born baby. In a sample of 2798 women living in Pennsylvania, USA, we tested our hypothesis using linear regression analysis of Shortened Postpartum Bonding Questionnaire (S-PBQ) scores, followed by longitudinal analysis using a generalized estimating equations model with repeated measures.

**Results:**

We found that women with a history of miscarriage had similar S-PBQ scores as women without a history of miscarriage at each of the three postpartum time points. Likewise, longitudinal analysis revealed no difference in the pattern of maternal-infant bonding scores between women with and without a history of miscarriage.

**Conclusions:**

Women in the First Baby Study with a history of miscarriage did not differ from women without a history of miscarriage in their reported level of bonding with their subsequently born infants. It is important for clinicians to recognize that even though some women may experience impaired bonding related to a history of miscarriage, the majority of women form a healthy bond with their infant despite this history.

## Background

The developmental process of becoming a mother is one of the most challenging experiences in a woman’s life [1] and although most women navigate the process successfully, a small percentage may develop impaired relationships with their infants. Maternal-infant bonding is one aspect of the developmental process that, successfully achieved, leads to a strong, healthy relationship between mother and infant. These healthy relationships promote infant growth and development as well as help to form a positive self-concept for the child later in life [2-4]. The concept of maternal-infant bonding is described in the literature as “an affective state of the mother; maternal feelings and emotions toward the infant are the primary indicator of maternal-infant bonding” [5]. Although impairments in maternal-infant bonding can lead to developmental disruptions for the child, and occasionally abuse and neglect, it remains unclear which sociodemographic, psychosocial, or other factors may place a woman at risk for impairments in maternal-infant bonding.

The developmental process of becoming a mother, and the many potential obstacles to this process, were described in detail in Ramona Mercer’s theory of Becoming a Mother (BAM) [6,7]. The theory generally describes the process of becoming a mother in four stages 1) commitment, attachment and preparation for the maternal role during pregnancy 2) acquaintance with and attachment to the infant, learning to care for the infant, and physical healing 3) moving toward a new normal, and 4) achievement of the maternal identity [8]. The theory provides some guidance for how impairments in the maternal-infant bond may develop, based on one key tenet of the theory: that a disruption in one stage of the process of becoming a mother will have a continued adverse effect on achievement of subsequent stages.

Miscarriage, defined here as an unexpected loss of pregnancy prior to completion of 20 weeks gestation, can be considered a disruption in the first stage of becoming a mother. The disruption may cause a negative effect on the mother’s perception of herself as a capable woman [9] and of her ability to successfully navigate the process when pregnant again subsequent to the miscarriage. Some evidence exists to support the idea that this disruption during pregnancy may affect all stages in the process of becoming a mother [9-11]. A mother with a previous history of miscarriage may therefore be at greater risk for difficulties navigating the process of becoming a mother and subsequently achieving positive maternal-infant bonding with an infant born subsequent to the miscarriage.

We found no research studies that specifically examined the relationship between a history of miscarriage and maternal-infant bonding. However, research examining the emotional impact of perinatal loss, which includes miscarriage, stillbirth, and neonatal death, provides some information to suggest that a history of miscarriage may negatively impact maternal-infant bonding. Two research groups [12,13] found that women with a history of perinatal loss reported lower attachment to their fetus during pregnancy than women without a history of perinatal loss. However, another study did not find a relationship between a history of perinatal loss and prenatal attachment [14]. Since prenatal attachment has been significantly correlated with postpartum bonding [15], it is reasonable to expect that women with a history of perinatal loss may have an increased risk of impaired maternal-infant bonding. Evidence also exists that women with a history of perinatal loss are more concerned with the health of their child [16,17] and report more problems with their child both in early infancy [18] and at school age [19]. However, Price [20] found no difference in the way mothers with a history of perinatal loss interacted with or perceived the behavior of their infants born subsequent to perinatal loss.

In a study of mothers of children born subsequent to stillbirth, Turton and colleagues [19] found that although mothers with a history of stillbirth reported that their children had more difficulties and peer problems than was reported by a control group of mothers, teacher ratings did not show any difference. This suggests that a relationship issue between the child and the mother may exist even when the child is otherwise perceived by others as functioning normally. Furthermore, studies by Hughes and colleagues [21] and Heller and Zeanah [22] showed that difficulties in the relationship between the mother and a 12 month old child born subsequent to perinatal loss could be explained by the mother’s mental representation of her prior perinatal loss. In other words, maternal thoughts about previous loss may interfere with the development of the mother-infant relationship. In a qualitative study of men and women parenting a toddler born after a perinatal or infant loss, Warland and colleagues [23] reported that parents distanced themselves emotionally from their subsequent child in order to protect themselves in case this child would also die. These studies all indirectly describe a disruption in the emotional relationship between the parent and a child born subsequent to perinatal loss. It is reasonable to hypothesize that these disruptions may begin in infancy with impairments in maternal-infant bonding.

As perinatal loss occurs in 12-20% of all confirmed pregnancies in the United States [24], and the majority of women will become pregnant again within 18 months [25], the effect of miscarriage on subsequent pregnancy and the subsequent maternal-infant relationship is of great concern. A history of miscarriage may affect not only women and their partners, but may also negatively affect the subsequently born healthy infant. The aim of this study is to examine the effect of miscarriage history on maternal-infant bonding after the birth of a healthy infant by longitudinally examining the relationship at 1 month, 6 months, and 12 months postpartum in a sample of women who have given birth to their first baby. Our hypothesis is that women with a history of miscarriage have decreased maternal-infant bonding at each time point compared to women without a history of miscarriage.

## Methods

### Study design and population

We completed secondary analysis of a multi-site longitudinal cohort study, the First Baby Study (FBS). Between January 2009 and April 2011, the FBS enrolled 3006 pregnant women planning to deliver their first live-born baby in the state of Pennsylvania. Women were excluded from the study if they did not speak English or Spanish, were carrying more than one fetus, had a previous stillbirth that occurred at more than 20 weeks gestation, had a previous cesarean delivery regardless of length of gestation, were a gestational or surrogate carrier, planned to give the baby up for adoption, planned to have a tubal ligation while hospitalized for delivery, did not have a telephone or were not able to commit to participation in the study for a period of 3 years. The study was approved by the Institutional Review Board at the Penn State Milton S. Hershey Medical Center and at participating study hospitals and written informed consent was obtained from each participant. A detailed description of the sampling design and recruitment plan is published elsewhere [26].

### Measures

Data were collected during telephone interviews at four time points. Sociodemographic and other background data were collected in the third trimester of pregnancy (baseline interview). Interviews were then conducted at 1 month, 6 months, and 12 months postpartum.

The independent variable, a history of miscarriage, was measured via self-reported history of miscarriage during a prior pregnancy. Women who were enrolled in the FBS and reported a history of elective abortion were excluded from analysis, and no other types of perinatal loss were present in the sample. Women with missing data at 1 month postpartum (n = 54) were also excluded from the present analysis. The resulting sample included 449 women with a history of one or more miscarriages and 2349 women with no history of miscarriage.

Sociodemographic variables (maternal age, marital status, race and ethnicity, education, and poverty status) were obtained during the baseline interview. Poverty was measured using the US Census Bureau classification system to categorize participants based on household income and family composition. Those with household incomes ≥ 200% above the threshold are classified as “not poverty”, those with household incomes that are 100% to 200% of the poverty threshold are “near poverty”, and those with household incomes < 100% of the poverty threshold are classified as “poverty”. For 127 women, regression methods were used to impute missing income values and create the poverty status category. Analysis completed with and without the imputed values revealed no difference in the results. Thus, imputed values were retained in the analysis.

Potential confounding variables including a reported use of fertility advice or treatment and history of anxiety or depression were also obtained during the baseline interview. Women were said to have used fertility advice or treatment if they had planned the pregnancy and responded affirmatively to the question, “Did you and/or your partner use any type of fertility advice, testing, or treatment before you became pregnant?” Women who reported that they had a doctor or nurse tell them that they had anxiety or depression prior to this pregnancy were considered to have a history of anxiety or depression.

At the 1-month postpartum interview, potential confounding factors of mode of delivery, infant hospitalization at birth, postpartum mental health visits, probable postpartum depression, and birth experience were measured. Probable postpartum depression was measured using the Edinburgh Postnatal Depression Scale (EPDS) [27]. Two of the original items were modified: “Things have been getting on top of me” was changed to “I have had trouble coping” and “The thought of harming myself has occurred to me” was changed to “The thought of harming myself or others has occurred to me”. Cronbach’s alpha for the EPDS in this study was 0.813. Participants were dichotomized as probable depression for EPDS >12 and not probable depression for EPDS scores ≤ 12, according to a systematic review [28]. Birth experience was measured using a 16-item scale with a potential range of scores from 16-80. A higher score indicates a more positive birth experience [29].

Potential confounding factors of maternal stress and social support were measured at each of the four time points and utilized as time-varying covariates. Maternal stress was measured using the *Psychosocial Hassles Scale*[30], an 11-item instrument which measures perceived maternal stress (from “no stress” to “severe stress”) due to common stressors, such as “money worries like paying bills”. We modified several of the items to fit the study population and added one item, “Problems with the baby”, for a total of 12 items. In this study, Cronbach’s alpha was 0.723 at 1 month postpartum and higher scores indicated higher levels of maternal stress. Social support was measured using 5 items from the *Medical Outcomes Study Social Support Survey*[31] and we added 4 items specifically concerning support for a new mother (i.e. “Someone to teach you what you need to know about taking care of a new baby” and “Someone to help you take care of the baby”). Cronbach’s alpha was 0.875 at 1 month postpartum and higher scores indicated higher levels of social support.

The outcome variable, maternal-infant bonding, was measured using a ten-item shortened version of the Postpartum Bonding Questionnaire (PBQ) which we called the S-PBQ [32,33]. The original PBQ is a 25-item screening questionnaire designed to identify women who are at risk for mother-infant relationship disorders [32]. A shortened version was created to address the need for an instrument to measure maternal-infant bonding within the constraints of a large telephone survey. Items for the S-PBQ were carefully chosen to represent each of the three original PBQ factors deemed adequate in sensitivity and specificity: Factor 1, impaired bonding, Factor 2, rejection and anger, and Factor 3, maternal confidence [32]. The S-PBQ measures bonding on a continuous scale with scores ranging from 10-50. Cronbach’s alpha in this study was 0.672 at 1 month postpartum. Further details about the scale development and psychometric properties can be found elsewhere [34].

With a large sample size of a total of 3006 women enrolled, the First Baby Study was adequately powered to detect small differences in general. Specifically, for our main variable of interest: maternal-infant bonding, the total number of usable sample was 2798 (with 449 with history of miscarriage and 2349 without). This enables us to detect an effect size (mean difference measured in the unit of standard deviation) as small as 0.17 with at least 91% statistical power [35].

### Analytic approach

Data analysis was completed using SPSS 20 and verified independently by the study statistician using SAS 9.3. First, Student’s t-tests were used to compare variables by miscarriage history. Second, univariate logistic regression models were built for each time point to examine the bivariate relationship between history of miscarriage and maternal-infant bonding. Then, multivariate linear regression models were built at each time point with adjustment for maternal age, use of fertility advice or treatment, marital status, race/ethnicity, education, poverty status, mode of delivery, infant hospitalization at birth, birth experience, postpartum mental health visits, history of depression, probable postpartum depression, maternal stress, and social support. The data were examined for potential violations of the assumptions of linear regression, and none were found except that S-PBQ scores were not normally distributed due to a negative skew. Normality was achieved using a reflected square root transformation of the data. However, the results of analysis using transformed data did not differ from the original analysis and therefore results using the original S-PBQ scores are presented for ease of interpretation. Finally, longitudinal analysis was completed using a generalized estimating equation model with maternal-infant bonding as a repeated outcome measure, adjusting for potential confounders maternal age, probable postpartum depression, and fertility treatment or advice.

## Results

Our participants were mostly married (72%), non-Hispanic White (85%), did not live in poverty (92%), and had completed a 4 year college degree or greater (58%). They had a mean age of 27.6 years. The characteristics of women in our sample by miscarriage history are shown in Table 1. Women with a history of miscarriage were on average 1 year older than those without a history of miscarriage (28.1 vs. 27.1 years, respectively, *p* < 0.001) and were more likely to have sought fertility treatment or advice prior to or during their pregnancy (20.3% of women with a history of miscarriage vs. 9.7% of women without a history of miscarriage, *p* < 0.001). Women with a history of miscarriage were also more likely to report symptoms of probable postpartum depression than women without a history of miscarriage (5.3% vs. 3.4%, respectively, *p* = 0.041). Women did not differ by miscarriage history on any other characteristics.

**Table 1:** Demographic and obstetric characteristics of study participants

	**Total**	**No history of miscarriage**		**History of miscarriage**		***p*-value**
	**N = 2798**	**N = 2349**		**N = 449**		
**Maternal age**	27.6 ± 4.3	27.1 ± 4.3		28.1 ± 4.3		<0.001***
**Marital status**						0.065
Married	2012 (71.9)	1673 (71.2)		339 (75.5)		
Not married	786 (28.1)	676 (28.8)		110 (24.5)		
**Fertility advice or treatment**						<0.001***
No	2480 (88.6)	2122 (90.3)		358 (79.7)		
Yes	318 (11.4)	227 (9.7)		91 (20.3)		
**Race/Ethnicity**						0.442
Non-Hispanic White	2370 (84.7)	1986 (84.5)		384 (85.5)		
Non-Hispanic Black	182 (6.5)	149 (6.3)		33 (7.3)		
Hispanic	144 (5.1)	127 (5.4)		17 (3.8)		
Other	102 (3.6)	87 (3.7)		15 (3.3)		
**Education**						0.828
High school graduate or GED or less	459 (16.4)	382 (16.3)		77 (17.1)		
Some college or vocational programs	725 (25.9)	613 (26.1)		112 (24.9)		
Completed 4 year college degree or greater	1614 (57.7)	1354 (57.6)		260 (57.9)		
**Poverty**						0.896
Poverty	230 (8.2)	192 (8.2)		38 (8.5)		
Near poverty	309 (11.0)	257 (10.9)		52 (11.6)		
Non-poverty	2259 (80.7)	1900 (80.9)		359 (80.0)		
**Mode of delivery**						0.115
Vaginal delivery	1981 (70.8)	1677 (71.4)		304 (67.7)		
Cesarean delivery	817 (29.2)	672 (28.6)		145 (32.3)		
**Infant hospitalization at birth**						0.089
No	2730 (97.6)	2297 (97.8)		433 (96.4)		
Yes	68 (2.4)	52 (2.2)		16 (3.6)		
**Postpartum mental health visits**						0.225
No	2667 (95.3)	2244 (95.5)		423 (94.2)		
Yes	131 (4.7)	105 (4.7)		26 (5.8)		
**History of depression**						0.132
No	2158 (77.1)	1824 (77.7)		334 (74.4)		
Yes	640 (22.9)	525 (22.3)		115 (25.6)		
**Probable postpartum depression**						0.041*
No probable depression	2695 (96.3)	2270 (96.6)		425 (94.7)		
Probable depression	103 (3.7)	79 (3.4)		24 (5.3)		
**Postpartum bonding score (S-PBQ)**	47.7 ± 2.6	47.7 ± 2.6		47.5 ± 2.7		0.261
**Birth experience**	68.7 ± 6.4	68.7 ± 6.4		68.5 ± 6.4		0.523
**Maternal stress**	15.5 ± 3.3	15.5 ± 3.3		15.5±3.4		0.780
**Social support**	38.9 ± 5.7	38.9 ± 5.6		38.7 ± 5.7		0.501

All results reported as n (%) or mean ± SD.

*p < 0.05, ***p < 0.001.

In univariate linear regression analysis, we found that miscarriage history was not significantly associated with maternal-infant bonding scores at each of the three postpartum time points (all *p* > 0.05) (Table 2). Likewise, when multiple linear regression analysis was completed, adjusting for variables that are theoretically related to miscarriage history and/or maternal-infant bonding, there was no statistically significant relationship between miscarriage history and maternal-infant bonding scores (all *p* > 0.05).

**Table 2:** Relationship between miscarriage history and S-PBQ score from linear regression models at 1 month, 6 months postpartum, and 12 months postpartum

	**Unadjusted model**		**Model adjusted for multiple factors‡**	
	**β (95% CI)**	***p*-value**	**β (95% CI)**	***p*-value**
**1 month postpartum:**				
No history of miscarriage	Ref		Ref	
History of 1 or more miscarriages	-0.15 (-0.41, 0.11)	0.268	-0.10 (-0.32, 0.13)	0.409
**6 months postpartum:**				
No history of miscarriage	Ref		Ref	
History of 1 or more miscarriages	0.02 (-0.19, 0.23)	0.858	0.04 (-0.16, 0.23)	0.725
**12 months postpartum:**				
No history of miscarriage	Ref		Ref	
History of 1 or more miscarriages	-0.01 (-0.24, 0.21)	0.907	-0.05 (-0.25, 0.15)	0.593

‡Model 2 adjusted for maternal age, use of fertility advice or treatment, marital status, race/ethnicity, education, poverty status, mode of delivery, infant hospitalization at birth, birth experience, postpartum mental health visits, history of depression, probable postpartum depression, maternal stress, and social support.

**Figure 1:**
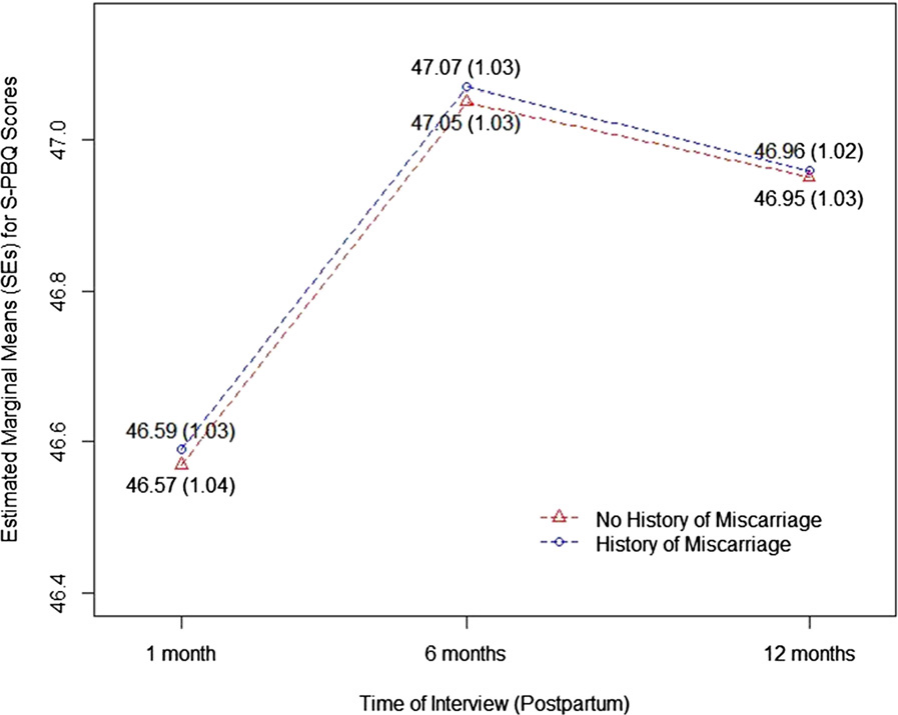
Estimated marginal means of S-PBQ score at each time point by miscarriage history using generalized estimating equations.

Longitudinal analysis revealed no statistically significant difference in the pattern of maternal-infant bonding scores over time between women with a history of miscarriage and those without. This is indicated by p-values greater than 0.05 for all miscarriage by time interaction terms (Table 3). In this analysis, a statistically significant relationship was revealed between interview time and maternal-infant bonding scores, as the lowest bonding scores occurred at 1 month postpartum and the highest scores at 6 months postpartum (Figure 1). This pattern of change over time in bonding scores did not differ by miscarriage history.

**Table 3:** Longitudinal relationship between history of miscarriage and S-PBQ score from generalized estimating equations with repeated measures

	**β(95% CI)**	***p*-value**
**Perinatal loss**		
No history of miscarriage	Ref	
History of 1 or more miscarriages	0.01 (-0.17, 0.20)	0.886
**Time**		
1 month	Ref	
6 months	0.48 (0.39, 0.57)	<0.001
12 months	0.37 (0.28, 0.47)	<0.001
**Age**	-0.03 (-0.05, -0.01)	0.001
**Fertility advice or treatment**		
No	Ref	
Yes	0.15 (-0.05, 0.35)	0.151
**Probable postpartum depression**		
No	Ref	
Yes	-2.45 (-3.01, -1.89)	<0.001

## Discussion

Our results indicate that women with a history of miscarriage report levels of maternal-infant bonding with their subsequently-born infants comparable to women without a history of miscarriage. This is in contrast to our original hypothesis that women with a history of miscarriage would have decreased maternal-infant bonding compared to women without a history of miscarriage.

Although previous studies have not specifically measured maternal-infant bonding in the context of a history of miscarriage, several have examined maternal reports of perceptions towards their subsequently-born children or maternal parenting behaviors in women with a history of various types of perinatal loss. In one study, mothers of 16-month-old children born subsequent to perinatal loss reported that they were more concerned about the child’s health, and more concerned with the psychological separation between mother and child as the child developed [17]. However, in another study, Price [20] reported that mothers with a history of perinatal loss did not perceive their 9 month old child as more difficult to raise than other children.

Hunfeld et al. [18] reported that mothers with a history of perinatal loss in their study were more likely to indicate that their healthy baby experienced problems with eating, sleeping, crying and acquiring a regular pattern of behavior compared to ideal babies at 4 weeks old. These researchers suggest that maternal perception of problems with the infant born subsequent to loss may be related to the comparison of the lost baby with the new baby. Since the lost baby was only an idealized child that didn’t cry or inconvenience the parent, the new baby may never be able to compare to the lost ideal baby. Although the results of extant research on parental perceptions of the child born subsequent to perinatal loss are mixed, there is some indication that maternal perceptions of the child may differ from those of women without a history of perinatal loss. The results of our study add to the current knowledge of the relationship between mother and infant born subsequent to miscarriage, as they indicate that a mother’s emotional response to the healthy infant, though it may differ from that of a woman without a history of miscarriage, leads to healthy maternal-infant bonding.

In terms of Mercer’s theory of Becoming a Mother, there are several possible explanations for our findings. First, it is possible that when a miscarriage occurs, it provides disruption in the first stage of the process of becoming a mother as we originally hypothesized, but that most women are able to cope in such a way that they achieve all four stages in a healthy manner during a subsequent pregnancy. Perhaps the successful completion of a pregnancy to full term gestation is enough to allow women the successful completion of stage 1 (commitment, attachment and preparation for the maternal role during pregnancy), such that the completion of stage 2 (acquaintance with and attachment to the infant, learning to care for the infant, and physical healing) is not negatively impacted. Another possible explanation is that for the majority of women, a miscarriage is not a significant disruption in the process of becoming a mother. Although most previous studies of perinatal loss, which were used to develop our hypothesis, did not differentiate between types of perinatal loss, Armstrong and colleagues [14] found that the gestational age of a previous perinatal loss was not significantly associated with negative emotional outcomes during subsequent pregnancy. It should be noted that we were unable to directly measure coping or a woman’s emotional response to her miscarriage in our study, rendering us unable to determine the exact explanation for our results in terms of Mercer’s theory.

The question that remains for researchers in this area of study is whether or not the alteration in perceptions of the child or overprotective parenting experienced by women with a history of perinatal loss is indeed a clinical concern that will affect the health of the mother or the child. Additionally, according to Price [20], studies in this area of research are often completed using a self-selection of participants based on their willingness to discuss previous perinatal loss experiences. This can create a selection bias where only women with the strongest emotional reaction to perinatal loss are included in the research study. In fact, in the only population-based study we found comparing women with a history of perinatal loss to those without on parenting outcomes, Price [20] found no association between perinatal loss status and observable measures of mother-infant interaction, or parental involvement with the child at 9 months of age. The only statistically significant difference by maternal perinatal loss history in Price’s study was that women with a history of perinatal loss reported that they sang songs and told stories to their infants more often than women without a history of perinatal loss. Our results concur with those of Price, for women with a history of miscarriage, and provide additional support for her assertion that the experience of parenting after a perinatal loss is a very individual and personal experience, and difficulties with maternal-infant bonding or other parenting tasks should not be assumed to be strongly influenced by perinatal loss history.

Our study has some limitations that deserve comment. First, the S-PBQ is a ten-item scale that is designed to measure a complex and multifaceted concept, maternal-infant bonding. Controversy exists in the literature regarding the most accurate way to measure maternal-infant bonding; however, our scale measures bonding in accordance with a recent concept analysis in which the authors concluded that measuring a woman’s emotional response to her infant, and not her behavioral response, is preferable [5]. Furthermore, the S-PBQ has not been widely used or validated in study populations other than this one, limiting the strength of our conclusions.

Additionally, women excluded from our sample due to missing data were younger, less likely to be married, non-Hispanic White, educated, and more likely to be living in poverty or depressed. As such, our results may be influenced by selection bias. There was also some selection bias due to differential loss to follow up such that mothers included at 12 months postpartum were older, more likely to be educated, married, non-Hispanic White, have postpartum depression, and less likely to live in poverty. However, those who were lost to follow-up did not differ from those included by miscarriage history and comprised a very small portion of our sample (6.4%). Also, our study participants were older, more likely to be non-Hispanic White, had higher levels of education and higher household income than the overall population of Pennsylvania [26]. These differences are common in longitudinal research studies where participation is voluntary, as healthy and well-educated women may be more willing to participate. As such, although our sample size was large and relatively diverse, the results of the study may not be generalizable to the all populations of women.

## Conclusion

In conclusion, we found no evidence that women in the FBS with a history of miscarriage show any difference in maternal-infant bonding with their subsequently born infant across the first year postpartum compared to women without a history of miscarriage. Although it is important for clinicians to recognize a history of miscarriage as a potential risk factor for disruption in the bond between mother and child, our study results indicate that clinicians should not assume that a history of miscarriage will definitely have a negative effect on the mother-infant relationship. Further research is necessary to determine the normative response to bonding with a healthy infant after a previous miscarriage. Future research should focus on the risk factors that may make women more vulnerable to a negative outcome related to a history of miscarriage. Additionally, future research studies should be designed using population-based samples if possible in order to reduce selection bias related to self-selection of women with a history of miscarriage.

## Abbreviations

EPDS: Edinburgh postnatal depression scale; FBS: First baby study; S-PBQ: Shortened postpartum bonding questionnaire.

## Competing interests

The authors declare they have no competing interests.

## Authors’ contributions

KHK planned the FBS study and completed data collection. CBK designed this secondary analysis, carried out literature research, and drafted the manuscript. CBK, KBR and JZ analyzed the data while KHK provided data management and oversight. All authors assisted with drafting and the critical revision of the manuscript and read and approved the final manuscript.

## Pre-publication history

The pre-publication history for this paper can be accessed here:

http://www.biomedcentral.com/1472-6874/14/83/prepub
